# Supplementary material for: Comprehensive Characterization of Lignans from Forsythia viridissima by UHPLC-ESI-QTOF-MS, and Their NO Inhibitory Effects on RAW 264.7 Cells
Source: Molecules. 2019 Jul 22;24(14):2649. doi: 10.3390/molecules24142649 (PMC6680392; doi:10.3390/molecules24142649)
Supplement: Supplementary file 1 [file molecules-24-02649-s001.pdf]

Supporting Information

Comprehensive characterization of lignans from *Forsythia viridissima* by UHPLC-ESI-QTOF-MS, and their NO inhibitory effects on RAW 264.7 cells

Jungmoo Huh [1,†], Chang-Min Lee [2,†], Seoyoung Lee [3], Soeun Kim[3], Namki Cho [3,*], Young-Chang Cho [3,*]

[1] College of Pharmacy, Seoul National University, Gwanak-gu, Seoul 08826, Republic of Korea; jmhuh112@gmail.com (J.H)
[2] Department of Laboratory Medicine, College of Veterinary Medicine, Chonnam National University, Gwangju 61186, Republic of Korea; cmlee1122@jnu.ac.kr (C.-M.L)
[3] College of Pharmacy, Chonnam National University, Gwangju 61186, Republic of Korea; kse3399@naver.com (S.K); cnamki@jnu.ac.kr (N.C.); yccho@jnu.ac.kr (Y.-C.C.)

† These authors contributed equally to this paper.
* Correspondence: cnamki@jnu.ac.kr (N.C.); yccho@jnu.ac.kr (Y.-C.C.) Tel.: +82-62-530-2925 (N.C.); +82-62-530-2925 (Y.-C.C.)

Figure S1. Chromatographic profiling of compounds in total extracts and fractions of *F.viridissima*.
Figure S2. Chromatographic profiling of compounds in subfractions of *F. viridissima*.
Figure S3. MS/MS spectra in positive mode of matairesinoside (**1**).
Figure S4. MS/MS spectra in positive mode of arctiin (**2**)
Figure S5. MS/MS spectra in positive mode of matairesinol (**3**)
Figure S6. MS/MS spectra in positive mode of arctigenin (**4**)
Figure S7. MS/MS spectra in positive mode of dimatairesinol (**5)**
Figure S8. MS/MS spectra in positive mode of viridissimaol A (**6)**
Figure S9. MS/MS spectra in positive mode of viridissimaol B (**7)**
Figure S10. MS/MS spectra in positive mode of viridissimaol E (**8**)
Figure S11. MS/MS spectra in positive mode of diarctigenin (**9**)
Figure S12. MS/MS spectra in positive mode of conicaol A (**10**)

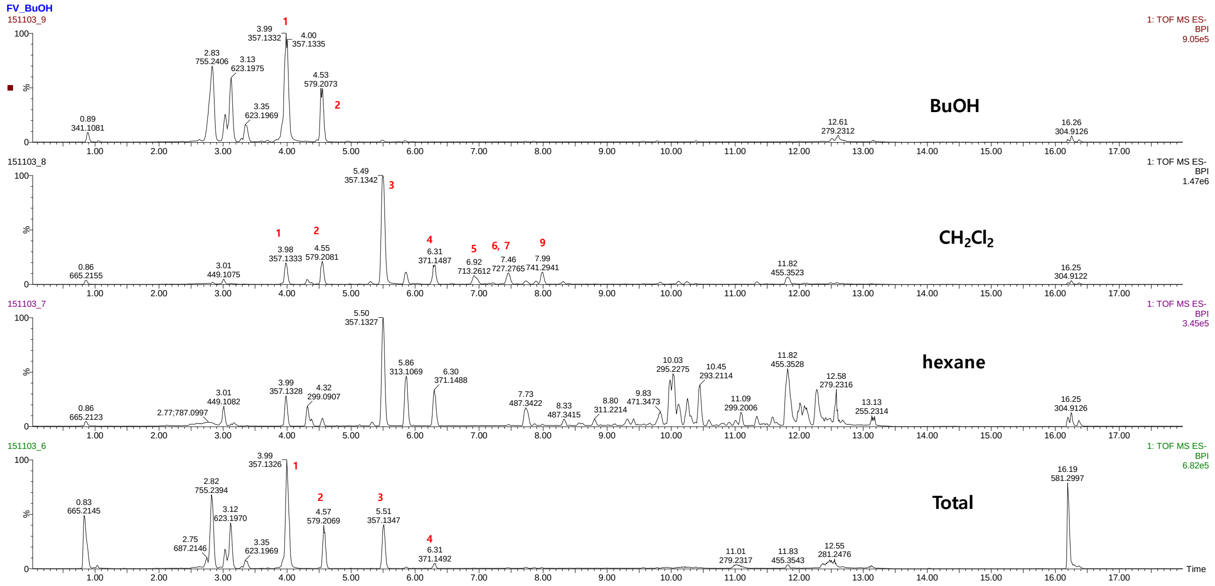

A: 0.1% formic acid water
B: Acetonitrile
Flow rate: 0.3 mL/min.

Figure S1. Chromatographic profiling of compounds in total extracts and fractions of *F. viridissima*.

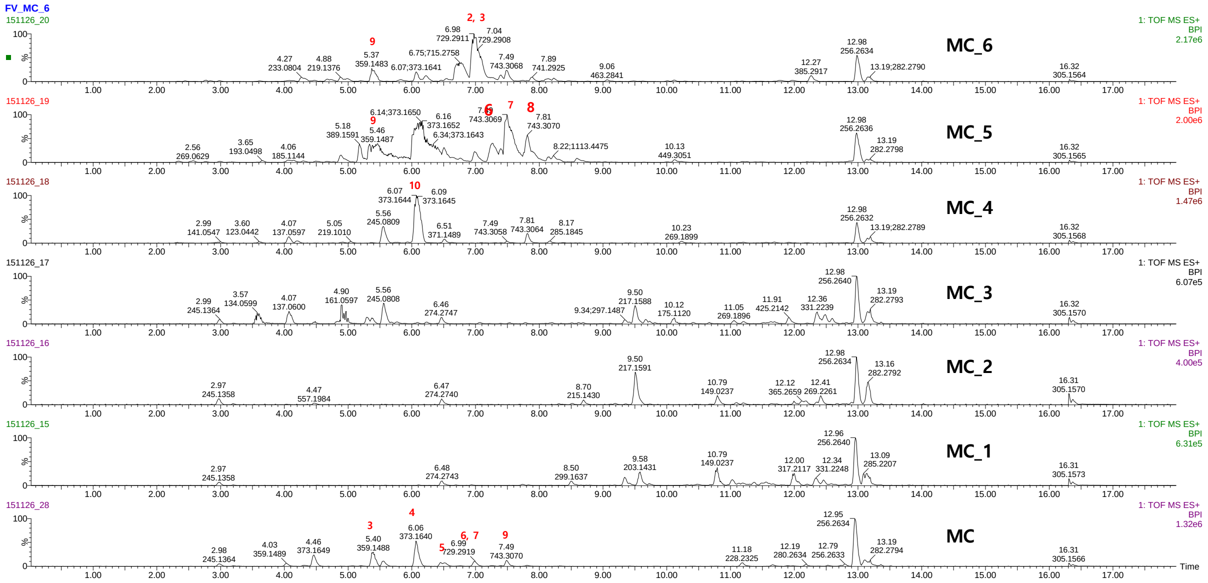

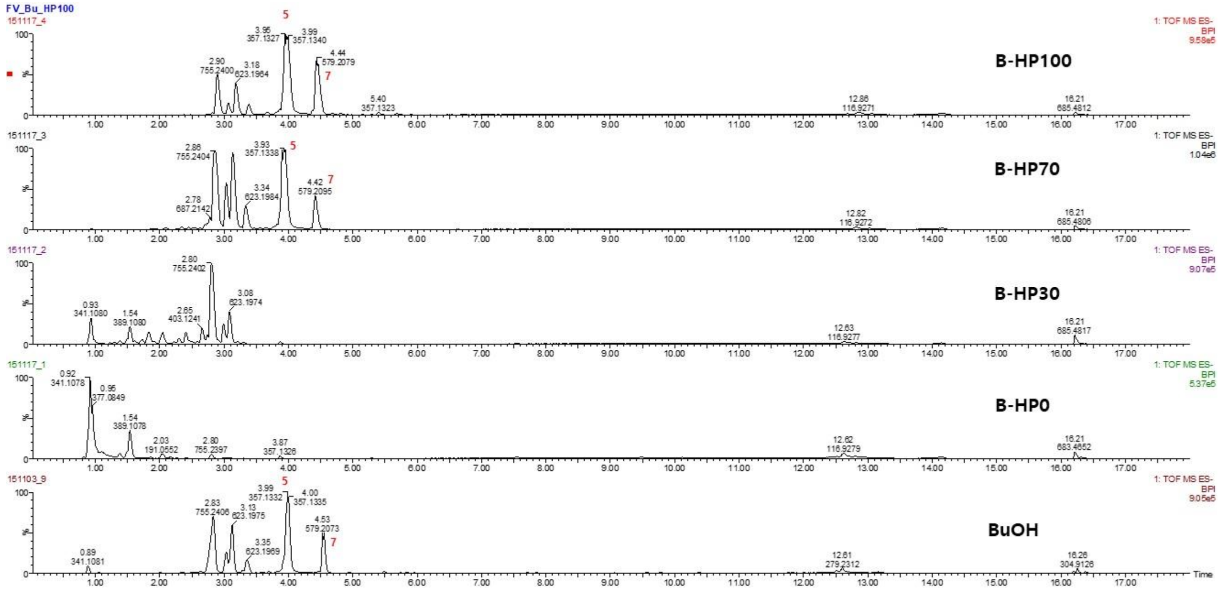

Figure S2. Chromatographic profiling of compounds in subfractions of *F. viridissima*.

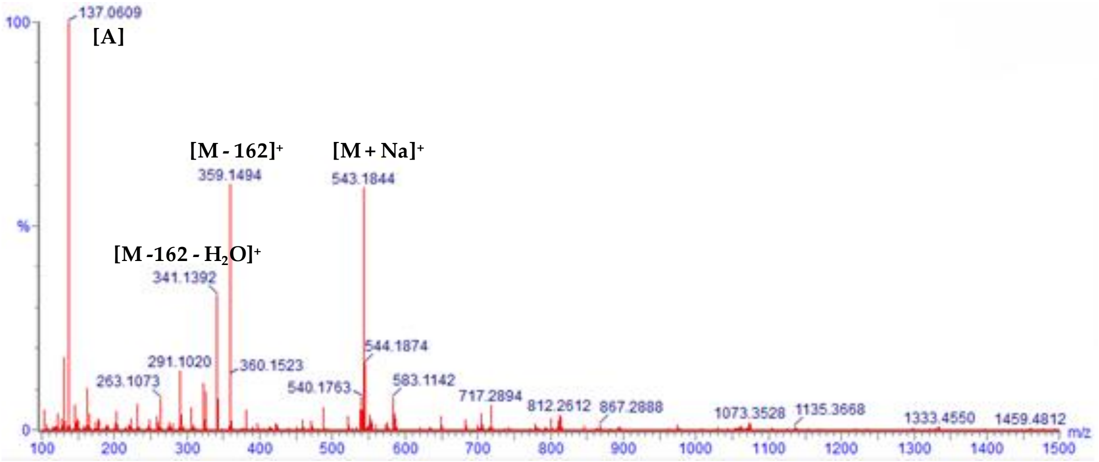

Figure S3. MS/MS spectra in positive mode of matairesinoside (**1**)

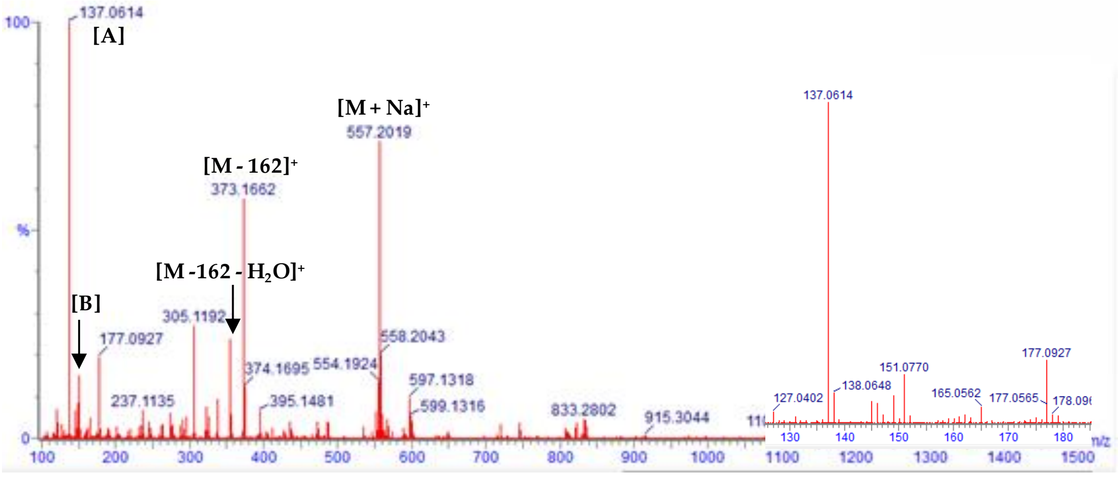

Figure S4. MS/MS spectra in positive mode of arctiin (**2**)

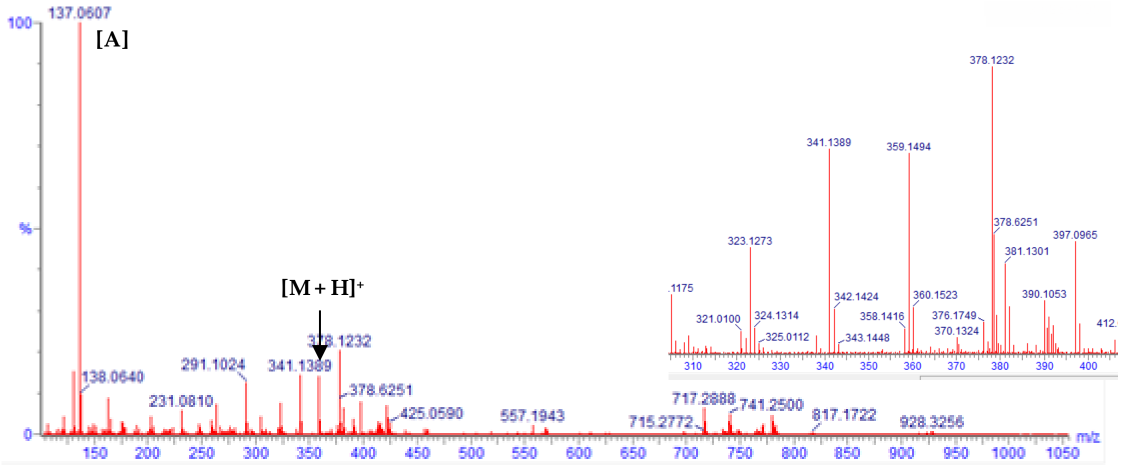

Figure S5. MS/MS spectra in positive mode of matairesinol (**3**)

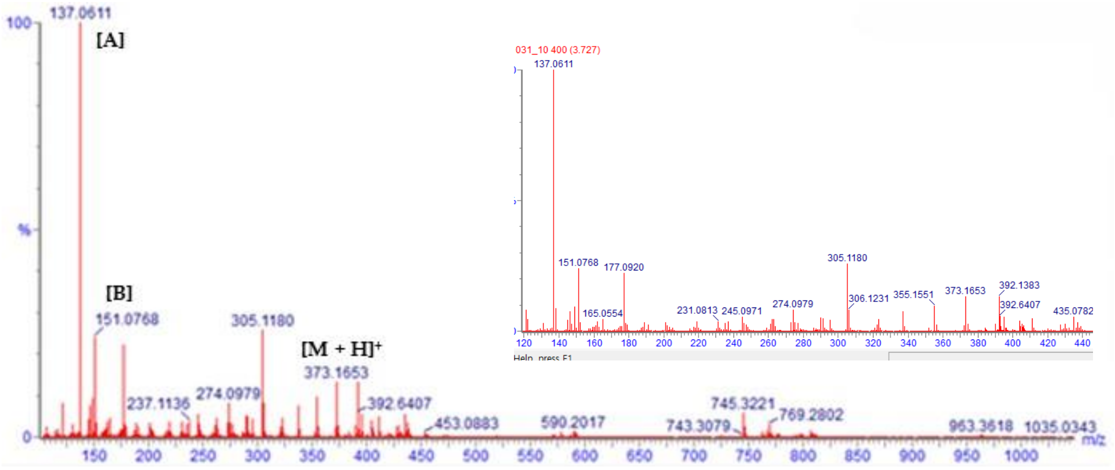

Figure S6. MS/MS spectra in positive mode of arctigenin (**4**)

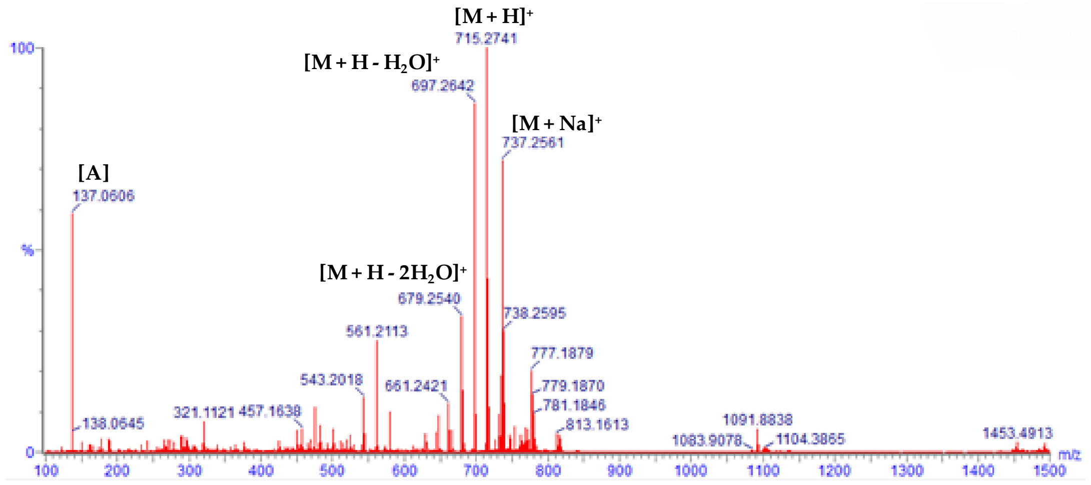

Figure S7. MS/MS spectra in positive mode of dimatairesinol (**5**)

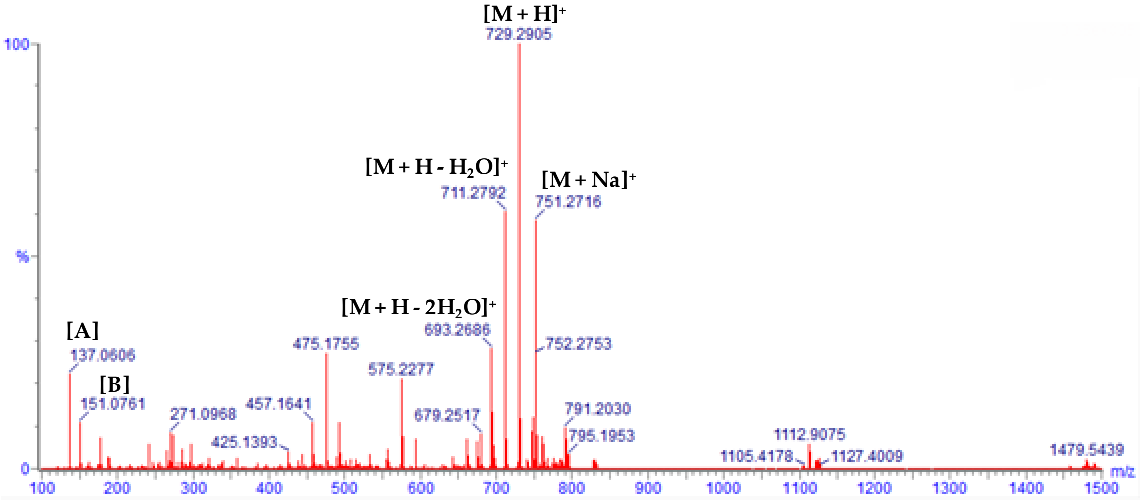

Figure S8. MS/MS spectra in positive mode of viridissimaol A (**6**)

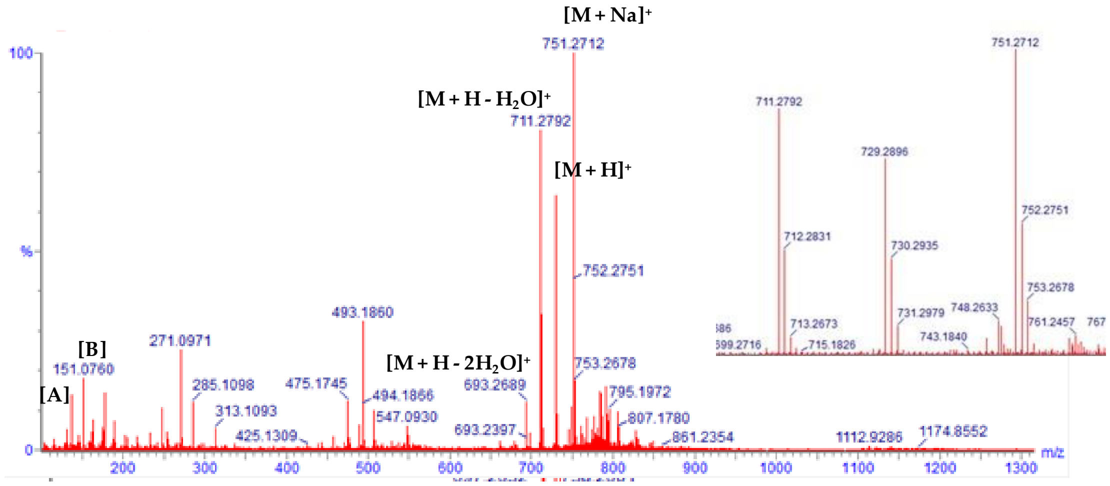

Figure S9. MS/MS spectra in positive mode of viridissimaol B (**7**)

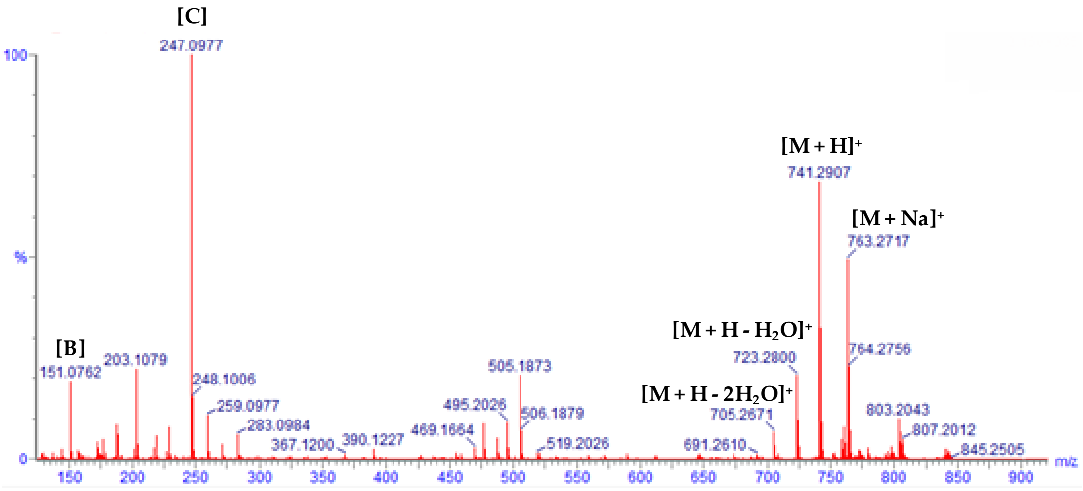

Figure S10. MS/MS spectra in positive mode of viridissimaol E (**8**)

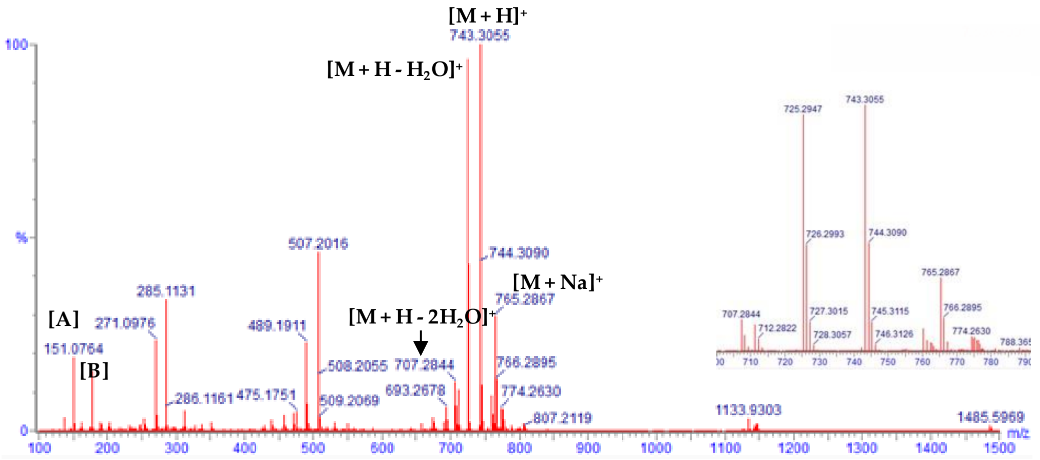

Figure S11. MS/MS spectra in positive mode of diarctigenin (**9**)

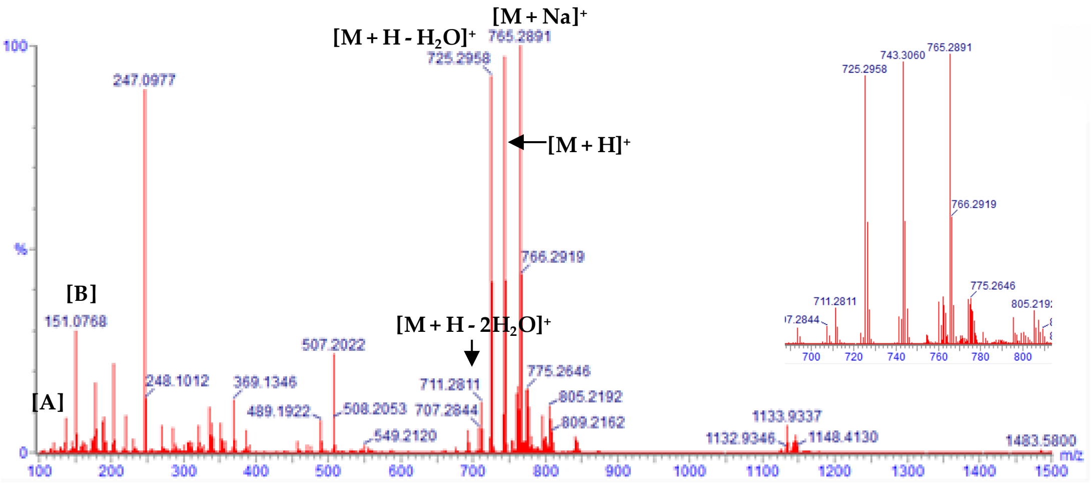

Figure S12. MS/MS spectra in positive mode of conicaol A (**10**)
